# Magnetic Resonance Imaging Features of Traumatic Patellofemoral Dislocation

**DOI:** 10.7759/cureus.3730

**Published:** 2018-12-14

**Authors:** Kunal Mohan, Prasad Ellanti, Marc Lincoln, Tom McCarthy

**Affiliations:** 1 Orthopaedics, Saint James's Hospital, Dublin, IRL

**Keywords:** acute, patella, dislocation, mri, radiographic, features, trauma, primary

## Abstract

Acute traumatic patellar dislocations are encountered with relative frequency, making up 3% of all knee injuries. Typically witnessed in younger patients following sporting injuries, this injury can be debilitating, potentially leading to recurrent dislocation, pain, reduction in activity and patellofemoral osteoarthritis.

Management of this injury remains controversial, and as such detailed magnetic resonance imaging (MRI) is increasingly recommended to help illustrate the exact nature of osteochondral and soft tissue injury, with a view to assessing the anatomical sequelae of patellar dislocation as well as the potential of recurrence and dictating the need for either conservative or surgical management in the acute setting. As such, awareness of the typical MRI findings in traumatic patellar dislocations may potentially aid in pursuing appropriate intervention for this pathology.

This case describes a 33-year-old gentleman presenting to the emergency department following patellar dislocation. After failed departmental closed reduction, this patient progressed on to definitive anatomical MRI assessment followed by acute surgical intervention in the form of medial patellofemoral ligament (MPFL) repair. This case allows for both illustration and discussion of typical radiological features associated with traumatic patellar dislocation.

## Introduction

Acute traumatic patellar dislocations are encountered with relative frequency, making up 3% of all knee injuries [1]. Typically witnessed in younger patients following sporting injuries, patellar dislocations generally occur laterally following either a twisting injury or direct impact, with resultant disruption of the medial patellofemoral ligament (MPFL), the primary restraint to lateral patellar subluxation, and accompanying retinaculum occurring in approximately 90% of affected patients [1,2]. While infrequent, this injury can be debilitating, potentially leading to recurrence in up to 40% with associated pain, reduction in activity and patellofemoral osteoarthritis [2].

Assessment of a primary traumatic patellar dislocation is multifaceted, involving initial clinical history and examination followed by initial plain radiographic evaluation involving anteroposterior, lateral and axial views [3]. While plain radiographs are useful in confirming the diagnosis, magnetic resonance imaging (MRI) is frequently recommended for definitive radiological evaluation following acute traumatic patellar dislocation, so to further assess soft tissue pathology and osteochondral injury and dictate the need for surgical intervention [2,3].

The following case report allows for illustration and discussion of some of the typical MRI findings one may encounter following acute patellar dislocation that may potentially precipitate subsequent definitive surgical intervention.

## Case presentation

**Figure 1:**
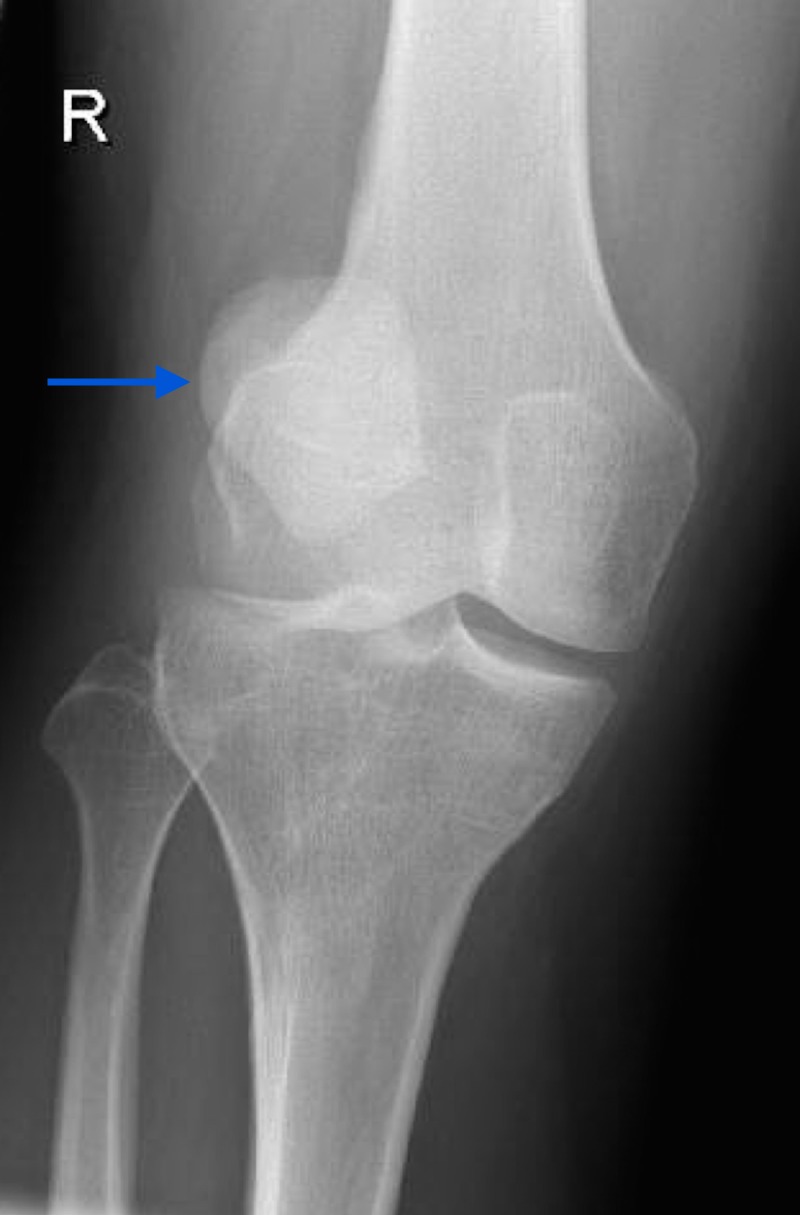
Plain anteroposterior radiograph of right knee showing lateral patellar displacement (blue arrow).

A 33-year-old gentleman presented to the emergency department describing substantial right knee pain and swelling following a rotational injury. Inspection indicated significant joint effusion and subsequent examination showed absence of straight leg raise on the affected side. Plain radiographic evaluation of the knee showed lateral displacement of the patella (Figure 1) confirming the clinical suspicion of a patellofemoral dislocation. Attempted closed reduction in the emergency department was unsuccessful, with the patella irreducible due to both an appreciable mechanical block and limited patient tolerance despite sedation. An MRI was thus performed to both assess any potential obstruction to closed reduction as well as to definitively evaluate the associated soft-tissue injury prior to determining the need for subsequent surgical intervention.

**Figure 2:**
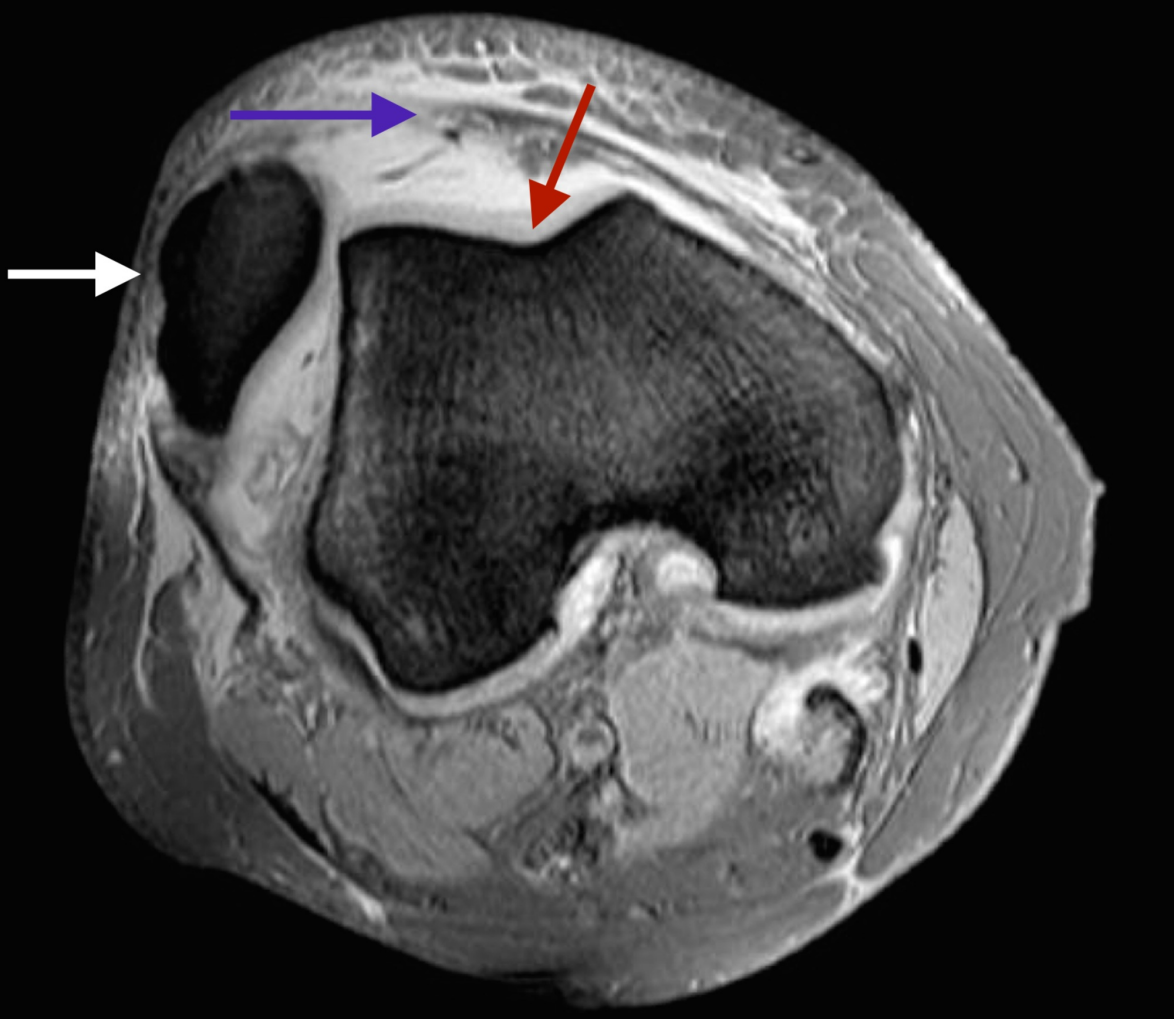
Axial magnetic resonance image of right knee showing lateral patellar dislocation (white arrow), bony avulsion of medial patellofemoral ligament (purple arrow) and a raised sulcus angle (red arrow).

**Figure 3:**
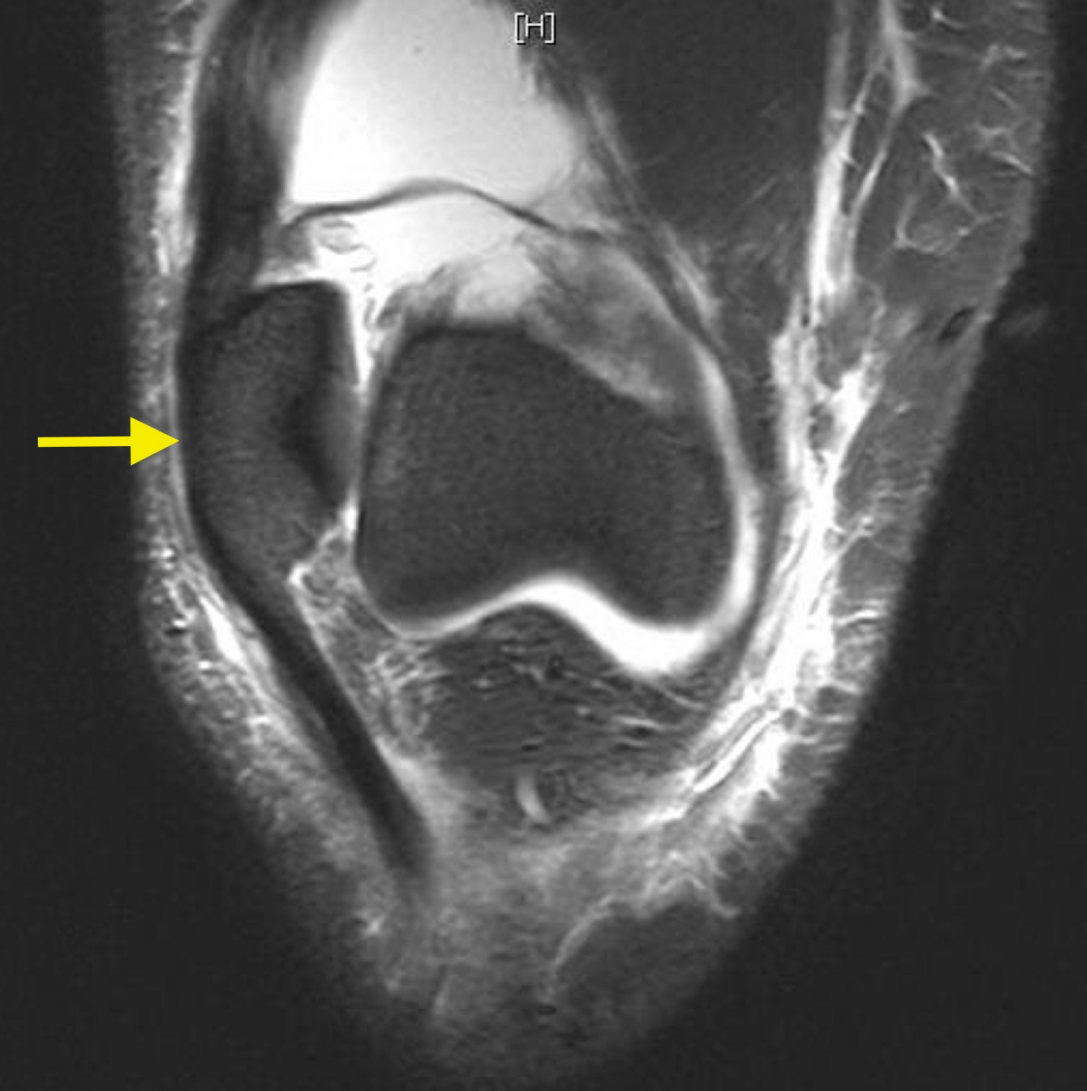
Lateralised, dislocated patella (yellow arrow) on coronal magnetic resonance image of right knee.

**Figure 4:**
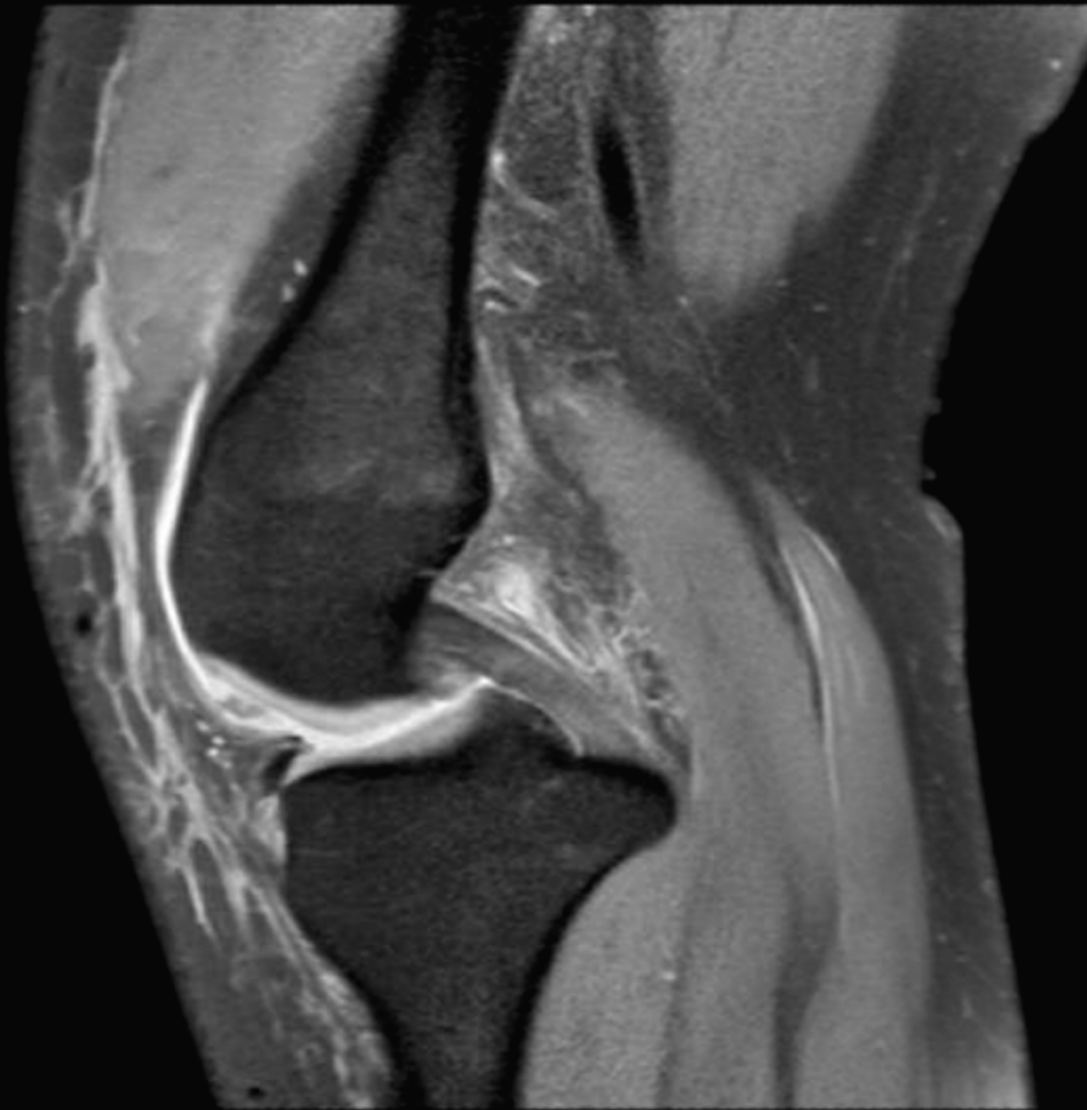
Sagittal magnetic resonance image of right knee.

Radiological investigations displayed evidence of lateral patellar dislocation on plain radiograph (Figure 1), as well as MRI features of traumatic patellar dislocation including patellar displacement, associated patellofemoral effusion, MPFL and medial patellar retinaculum tears and avulsed patellar cartilage (Figures 2-4). Additional MRI assessment showed a raised sulcus angle (>140) (Figure 2).

In the context of failed closed reduction as well as confirmation of the diagnosis, associated soft-tissue injuries and presence of risk factors for recurrent dislocation, this patient subsequently proceeded to definitive surgical treatment in the form of MPFL repair, with excellent postoperative function and no further dislocation at 46 months postoperatively.

## Discussion

Acute patellar dislocations are debilitating, with up to 58% of patients noting persistent limitation to strenuous activity and 55% failing to return to sport at six months following index injury [3,4]. While the majority of patients do not suffer from recurrent dislocation, those suffering from recurrence are at higher risk of persistent symptoms as well as patellofemoral osteoarthritis [2,3,5]. As a result, management of acute patellar dislocations in an attempt to address the risk of recurrence in the acute setting remains under debate, with a recent increase in acute surgical management occurring with potentially more favourable functional status, recurrence and complication rates but conservative management continuing to show acceptable long-term outcomes [3,6].

Detailed anatomical illustration of the patellofemoral joint and surrounding structures at time of injury has thus become increasingly important aspect of initial evaluation following this injury [6-8]. This is typically achieved through MRI, which has an increasingly prominent role in helping dictate the definitive decision on nonoperative versus operative treatment in acute patellar dislocations [2]. MRI is particularly useful in detecting diagnostic factors of patellar dislocation, namely joint effusion, cartilaginous contusions of the lateral femoral condyle and medial patella, the presence of osteochondral lesions as well as the characteristically associated MPFL and medial retinaculum tears [1]. Additionally, it allows for surveillance of concomitant knee pathology. While a patient specific judgement, the presence of osteochondral injury as well as the existence of risk factors for recurrent dislocation, which include MPFL injury, raised sulcus angle, trochlear dysplasia, raised tibial tuberosity-trochlear groove distance, and an increased Insall-Salvati Index, pathognomonic of patella alta, each of which is assessed on MRI with high levels of specificity may indicate that surgical intervention is warranted [1-3,9].

Given both the increased diagnostic role of MRI and the relative frequency in which this injury is encountered, particularly in the adult population, awareness of both the plain radiographic features and more importantly the MRI findings associated with acute traumatic patellar dislocation as discussed above may aid in improved detection, earlier management and prevention of recurrent dislocations, hence reducing the potential debilitating effect of this injury [1,2].

## Conclusions

Traumatic patellar dislocations most commonly occur laterally and are associated with injury to both the medial patellofemoral ligament and medial patellar retinaculum. MRI is becoming an increasingly important diagnostic tool in assessment of traumatic patellar dislocations, useful in both detecting associated cartilage and soft tissue disruption as well as risk factors for recurrent dislocation. As such, awareness of the typical MRI findings in traumatic patellar dislocations outlined in the above case may potentially aid in pursuing either definitive conservative or surgical treatment in this pathology.
